# The role of personal purpose and personal goals in symbiotic visions

**DOI:** 10.3389/fpsyg.2015.00443

**Published:** 2015-04-14

**Authors:** Jodi L. Berg

**Affiliations:** Doctor of Management Programs, Weatherhead School of Management, Case Western Reserve UniversityCleveland, OH, USA

**Keywords:** higher purpose, calling, meaning, vision, shared vision, motivation, engagement, relationships

## Abstract

It is believed that symbiotic visions can drive employees and organizations toward a common objective based on the premise that people have a high level of self-motivation and engagement when they are working toward something very personal. The field of organizational development has been aspiring to help organizations and people align their visions for decades without much, if any, empirical support for the role of personal purpose and goals in the symbiotic relationship with a company vision. This qualitative study examines the role personal purpose and goals play in how high performing leaders align to their company's vision. Whether and how senior managers articulate this alignment, and its correlation to their motivation and engagement, was examined. An observation was that most senior managers within organizations with a well-developed and widely known higher purpose vision are driven by something personal, identified as either personal goals or a personal purpose. One of the key findings is that personal purpose and goals, when aligned to a company vision, appear to impact motivation and engagement in different ways. When alignment is felt through the sense of the greater purpose, there is a deep, almost spiritual, commitment to making the world a better place and helping the organization contribute to that. This seems to motivate them to guide the organization toward its higher purpose vision. When alignment is felt through the organization's alignment to one's personal goals, there is a great sense of commitment to completing the steps or tasks necessary to move toward the vision, yet a clear delineation between work and life ambitions.

## Introduction

Certain people appear to be motivated and engaged in achieving an aspirational purpose or personal goal such that it aligns their efforts, their thinking, and their decision making. The contemporary research on the topics of purpose, company vision, motivation and engagement is prolific. Duffy and Dik (2013) referenced approximately 40 recent studies on the topic of purpose and calling and the connection to work-related and general well-being (Duffy and Dik, 2013). Also, there are numerous practitioner books on using purpose, calling, and vision to reach the heart of an employee, such as *Purpose and Meaning in the Workplace* (Dik et al., 2013).

Yet, even if individuals are externally motivated by a company's vision and are working in a stimulating environment, they are still unlikely to experience the intrinsic motivation, engagement, and fulfillment that comes from working toward the accomplishment of one's own personal ambition (Boyatzis and Akrivou, 2006). Consider that often, at work, one is coached to comply with someone else's vision (Boyatzis et al., 2012); working hard to be the underling their boss wants them to be in order to contribute to the goals of the organization. Instead of advancing toward one's personal definition of who they want to become, their ideal self, employees are working toward an “ought self,” or the understanding of what one should be based on someone else's vision (Boyatzis and Akrivou, 2006). Through the development of the self-determination theory and the need for competency and autonomy, Deci and Ryan (2000b) contributed to understanding motivation in the social context; explaining that experiencing motivation and engagement by embracing someone else's vision may very well work for a while. Boyatzis and Akrivou (2006) advanced the understanding by discovering that one can be perfectly content working toward someone else's goal or objectives until one realizes that their personal dreams are being compromised because this “ought self” does not match their ideal self. This awakening leads to feelings of betrayal and frustration for having wasted energy pursuing the dreams and expectations of others. This creates what Boyatzis (2008) called negative emotional attractors (NEA) which have an adverse effect on motivation and engagement (Boyatzis and Akrivou, 2006).

This study is timely for businesses because employee commitment to the organization, connection to its purpose and engagement at work are all cited as major motivators of people staying in a company (Ashforth and Mael, 1989; Kapoor and Meachem, 2012). From the individual's perspective, it has long been identified in the literature that the work one does is significant to a person's sense of meaning and identity. In *Purpose and Meaning in the Workplace*, the editors pull from work all the way back to Adler in 1931 and Erikson in 1963 to support their statement that, “Along with love, play, and community, work at its best offers a core context for construction of self and contributing to society in ways heartfelt, personally meaningful and socially relevant” (Dik et al., 2013, p. 17). Yet, there is limited empirical support for the role one's personal ambitions play in the symbiotic relationship to a company's higher purpose vision.

## Research question

This research is about understanding the roles personal purpose and goals play in the symbiotic relationship with their company's vision. To be able to understand personal purpose and goals it was important to minimize the other variables. Because it would be expected that high performers are already motivated and engaged, interviewing high performers allowed me to focus on the nature of personal purpose and goals without the added complexity of whether motivation and engagement exist. The research question for this study is: What are the roles of the personal purpose and goals of high performing leaders in symbiotic relationships with their company's higher purpose vision?

## Literature review

The understanding of purpose has evolved quite significantly over the past 60 years. Prior to the 1959 publication of Frankl's book *Man's Search for Meaning*, purpose and meaning were understood to be a way of adapting and not as a motivator toward change (Damon et al., 2003). Frankl was the first to identify purpose and meaning as more than derivatives of motivation by recognizing them as the drivers used to overcome circumstances (Frankl, 1959).

Personal purpose, meaning and calling are often interrelated (Elangovan et al., 2010), and/or used interchangeably with each other (Boyatzis and Akrivou, 2006; Duffy and Dik, 2013). Elangovan et al. (2010) indicates that purpose and calling hold many of the same attributes, such as hope and a focus on others, based on an inner desire to stay true to oneself and to do the right thing, make the world a better place or pursue a well-meaning goal (Elangovan et al., 2010). When working toward a “calling,” one receives personal gratification (Novak, 1996) and a sense of personal purpose (Hall and Chandler, 2005). It is personal and the tasks are more enriching (Cardador et al., 2011), which is intellectually and emotionally stimulating (Bakker et al., 2008). McKnight and Kashdan (2009) describe purpose as a “centralized, self-organizing life aim that organizes and stimulates goals, manages behaviors, and provides a sense of meaning” (p. 242). Whereas, a calling is thought to be bestowed upon one by a higher power or a response to a strong inner passion, and a purpose is discovered or found, calling and purpose both drive action and define one's identity (Elangovan et al., 2010).

Recent research has made some progress deconstructing and discerning meaning and purpose. Rainey cited much of the research already referenced as well as work by Heintzelman and King (2014), Hicks, Cicero, Trent, Curton, and King (2010), and Rockind (2011) to conclude that the “two are distinct phenomena that differ in their orientation toward cognition or action and in their temporal framing. Meaning and purpose are separate, albeit highly related, constructs that build off of one another so as to contribute to the broader concept of the ‘good,’ or meaningful life” (Rainey, 2014, p. 22). This deconstruction connects meaning to an integration of the past, present, and future whereas purpose is a future directed element of meaning that may not integrate past and present (Baumeister et al., 2012).

In addition to being future directed, the construct of purpose is becoming more understood to include doing something that a person feels driven to do in which the benefactor or benefactors are not themselves (Duffy and Sedlacek, 2007). Damon et al. (2003) characterized a sense of purpose as denoting a course that is personally meaningful and beneficial to the greater society.

In this study, I sought to explore the impact of purpose, separate from both a calling from a higher source and making meaning through an integration of past experiences, while still holding to the elements of purpose that appear to have consistently emerged in psychological literature. For this study, I defined personal purpose as: *a deliberate choice to pursue a future directed intention that is personally meaningful, and beneficial to the greater society, that influences one's goals and behaviors*.

### Motivation and engagement

Engagement and motivation often go hand in hand (Kapoor and Meachem, 2012; Tillott et al., 2013). In this study, motivation and engagement were both selected as variables because they appear to be relevant to achieving personal aspirations, but in different ways. In existing research, motivation is connected to determination, while engagement is linked to a state of awareness. Motivation is what creates action and drive (Berlyne, 1964), whereas engagement is the state of mind and energy committed to that action and accomplishment (Kapoor and Meachem, 2012).

Motivation has been associated with harnessing personal drive in several theories developed over time. Maslow's need for self-actualization created a foundation for personal motivation with his statement that, “What a man can be, he must be” (Maslow, 1943, p. 380). This statement infers that self-actualization drives one to work toward what one “could” be. Herzberg (1968) made a big contribution to the understanding of motivation by introducing the contrast between extrinsic (e.g., external rewards) and intrinsic motivation (e.g., personal sense of achievement or connection with the work itself). With the exception of the external impact of relationships, this study is primarily about intrinsic motivation.

It appears that the more personal the accomplishment, or intrinsic the center of causality, the higher the motivation to excel. Mallett and Hanrahan applied the social-cognitive theory of motivation in the context of elite athletes. In their study, they concluded that elite athletes “were highly driven by multiple personal goals and, in particular, self-determined motivation” (Mallett and Hanrahan, 2004, p. 198). They found a noticeable difference in the effort and drive in athletes that wanted to succeed because of personal ambition vs. athletes that were driven by external forces and rewards. In essence, the more the locus of causality is internal, the higher the degree of self-determination (Mallett and Hanrahan, 2004).

The understanding of engagement has evolved over time as well. In developing his construct of personal engagement and disengagement, Khan built upon existing literature on “person to role” relationships done by Lawler and Hall 1970, Lodahl and Kejner, 1965, Mowday et al., 1982, Porters et al., 1974, Blauner, 1964, and Seeman 1972 (Kahn, 1990). He articulated the terms *personal engagement* and *disengagement* to “refer to the behaviors by which people bring in or leave out their personal selves during work role performances” (Kahn, 1990, p. 694). The term *engagement* was later defined by Harter, Schmidt and Hayes as a person's involvement in and satisfaction with their work (Harter et al., 2002). A *high* level of engagement is the result of being stimulated, positive and fulfilled with a strong sense of meaningful pursuit and dedication (Bakker et al., 2008). Engagement was defined by Bakker et al. (2008) as a positive and pleased state of mind, categorized by vigor, commitment, and captivation, commonly understood to generate higher levels of energy and a strong connection to work. Boyatzis, Smith, and Beveridge also connected engagement with increased energy, focus and drive through their research on “Positive Emotional Attractors.” They validated this theory by linking PEA to physical stimulation—identifying the physiological activation that occurs during the actual experience of an elevated state of engagement, hopefulness, and future orientation. When reaching for a personal vision one is engaged, emotionally and physically, in moving toward an overarching goal. The goal becomes meaningful and purposeful enough to impact their energy, their focus and their drive (Boyatzis et al., 2012). This is also supported by the evidence that the desire to achieve one's “ought self,” or the self that we feel we ought to be, is less than the desire to reach for our ideal self. When we are working to accomplish a goal or vision that is not our own, we are less driven (Higgins, 1987; Boyatzis, 2008).

This is relevant because it exposes a gap in potential engagement and motivation when the vision or purpose individuals are striving to achieve belong to someone else (e.g., another person or an employer). This is relevant because it illustrates there may be a difference between the individual who is striving to achieve a company vision that is not their own, and the individual who is striving to achieve their personal purpose through the work they do for an organization.

### Connecting purpose to motivation, engagement, and performance

There is a strong desire to embrace purpose as illustrated by the popularity of Rick Warren's book *The Purpose Driven Life*, which by its tenth anniversary had sold more than 60 million copies. The connection of purpose to motivation, engagement and performance is already established. The positive psychology movement that studies the flourishing aspects of psychology connects purpose to motivation, engagement and performance, recognizing both purpose and calling as sources to motivation, drive toward, and commitment to, an accomplishment (Seligman and Csikszentmihalyi, 2000; Damon et al., 2003). Embracing a calling, purpose or personal vision in one's vocation, as well as the feeling of living out a calling, is linked to a positive work experience and well-being (Duffy and Dik, 2013). And an increased level of meaning or purpose is connected with work gratification (Bonebright et al., 2000), life fulfillment, well-being (Zika and Chamberlain, 1992) and happiness (Debats et al., 1993). Organizations in which employees experience a higher level of engagement have increased levels of performance than organizations that do not (Macey and Schneider, 2008). Shuck and Rose (2013, p. 343) take this even further by discovering that “engagement and performance are a secondary consequence to work that is interpreted as meaningful and purpose-driven.”

### Company vision and its connection to motivation, engagement, and performance

The definitions of company vision vary but they all contain elements of an ideal future state. Vision is a desired state of products, services and an organization that a leader wants to realize (Bennis and Nanus, 1985). It is an idyllic and distinctive representation of the future (Kouzes and Posner, 1987). Vision is a desired state that represents or echoes the collective values of an organization (House and Shamir, 1993). A vision helps define why people should, and how people will, act with regards to performance, decisions, and dealing with conflict (Reilly, 2008).

Empirical evidence about the connection between vision and performance is mounting. CEOs with a vision significantly outperformed CEOs that were not leading their organization with a vision (Baum et al., 1998). A study of mergers and acquisitions (M&A) demonstrates that vision is a critical factor leading to successful performance, particularly during initial phases of M&A when companies are expected to recover acquisition costs and generate a return on investment (Clayton, 2009). Neff demonstrated the positive correlation between vision and the success of family businesses (Neff, 2011). Vision was also highlighted as a determining factor that enables daughters to overcome gender bias and become successors in family businesses (Overbeke, 2010). A longitudinal study by Baum et al. (1998) found a causal effect of vision and vision communication on organizational-level performance. The directional effects in their study support that vision is an impactful factor in company performance (Baum et al., 1998).

Vision drives motivation (Mirvis et al., 2010) and is connected to both motivation and engagement through the work of Boyatzis and Akrivou (2006) who validated that vision, at both the individual and organizational levels, is a key element in successful, sustainable change which drives engagement and meaning.

Even more effective is a shared vision which allows for the incorporation of different perspectives within the organization, creating buy-in and support (Kapoor and Meachem, 2012; Tillott et al., 2013).

The concept of a personal higher purpose, introduced by Duffy and Sedlacek (2007) as one in which the benefactors are not themselves, could be applied to organizations. The concept is that a higher purpose vision positions a company to build a financially sustainable organization that creates both social good (e.g., making the world a better place) and social capital (e.g., trusting and committed relationships with all stakeholders) (Beer and Norrgren, 2011). A higher purpose goes beyond generating only profits and shareholder value (Mackey and Sisodia, 2014).

### Relationships

Relationships are very relevant to this study on the personal, social, and organizational level. According to Van Oosten (2006), supportive and trusting relationships are the fulcrum that allows change to take place. Positive relationships are correlated with a greater degree of engagement, commitment, and retention (Dirks and Ferrin, 2002). In the intentional change theory, relationships actually facilitate the movement through each discovery that brings about purposeful change. A correlation was identified between the pursuit of the ideal self and the physiological effect it has on neural circuits, appetite for learning and the emotional state of elation; all increasing the level of engagement around one's dreams, hopes, and strengths (Boyatzis and Akrivou, 2006).

Social identity and its correlation to relationships appear to be important in the work environment. Social identity is defined by Ashforth and Mael (1989) and Boyatzis and Akrivou (2006) as the relationships one has with groups with which one is connected. Tajfel (1982) began his early work on social identity theory in the 1960s through the identification of one's self to his or her group memberships. Although the initial context was to explain the tendency to elevate one's self image by identifying with groups or categories, the framework has been extended. Boyatzis and Akrivou validated the connection between group relationships to elements of individual performance and organizational direction, beyond the original connection to self-elevation.

The intentional change theory establishes that positive, energizing relationships are not only critical in supporting change but a sense of group identity is an important element in the construct of shared vision (Boyatzis and Akrivou, 2006). Identification with the social identity of an organization facilitates the internalization of company values and beliefs (Ashforth and Mael, 1989). Ashforth and Mael referenced research already done on social identities within the organizational framework when they wrote that, “Organizational identification has long been recognized as a critical construct in the literature on organizational behavior, affecting both the satisfaction of the individual and the effectiveness of the organization (Brown, 1969; Hall, Schneider and Nygren, 1970; Patchen, 1970; Lee, 1971; Rotondi, 1975; O'Reilly and Chatman, 1986)” (Ashforth and Mael, 1989, p. 20).

The literature illustrates that the constructs of personal purpose, company vision, motivation, engagement, and relationships have been emerging over time but there appear to be enough common elements in their definition to use as a foundation for gaining a greater understanding of the nature of personal purpose and goals when a company's vision is symbiotic with the aspirational purpose or goals of the individuals.

## Method

I am exploring the role purpose and goals play in symbiotic relationship with a company vision, so a qualitative approach was used, allowing for an abstract theoretical exploration of the social experience (Charmaz, 2012).

A cyclical process of gathering data, coding, reflection and review through memos was used to allow theoretical ideas, categories, and themes to emerge. The themes were then examined for validity against the codes.

Data was gathered through interviews with open ended questions; allowing the interview process to be flexible, and the conversation to flow and evolve. The interviews were designed to pull out stories and personal experiences that illustrated connections between personal purpose, goals, corporate vision, positive relationships, engagement and motivation. Understanding that one's personal purpose is often evolving and changing, the questions were intended to capture the connections between their understanding of their personal purpose or goals at that time. The questions used during the interview process are listed in the Appendix—Supplementary Materials.

Because the literature indicated that even in the academic world the words purpose, meaning, calling, and driver are often used interchangeably and are considered interrelated, I was careful not to focus on just the word “purpose” during the interview.

The research was done in the context of high performing, senior leaders in U.S., for-profit organizations. Because I wanted to observe the way people articulated the role personal purpose and personal goals played in the alignment to, or possibly a symbiotic relationship with, a company's higher purpose vision, I selected a group that would most likely already be aligned to their company's vision.

I sought out companies that had a higher purpose statement that was communicated publicly. A higher purpose statement was defined as an articulated vision statement that is future directed and beneficial to the greater society. Companies with a published higher purpose statement were identified through personal networks and publications that recognized businesses as having a higher purpose vision. The initial quantity of possible candidates was large to assure there were enough companies that fit the criteria. Each recommendation was vetted to meet the definition of higher purpose by researching the company online, reviewing company literature, as well as asking people within the industry, and within the organization, to determine if the company indeed had a higher purpose that was shared within the company.

To assure a large enough pool of people to be interviewed, it was important that each company had multiple layers of management, enough breadth to include more than six high performing leaders, and an established performance review process. The organizations in which I conducted interviews all had annual sales revenue above $600 million.

I conducted interviews within four companies that met the criteria, and provided geographical and industry diversity. I was introduced to the CHRO, company president or divisional vice-president by mutual acquaintances. They connected me with an internal resource who identified members of the senior leadership team considered to be high performers. High performers were defined as individuals who had received an “exceeds expectations” or the equivalent rating on their performance reviews as established within the individual organizations, or were identified as high performers by their supervisor.

One of the outcomes of the goal-setting theory is that goals refer to important future outcomes and therefore, the selection of goals infers a desire to achieve a purpose or consequence; and success is associated with one's ability to pursue and accomplish goals that are important and meaningful (Locke and Latham, 2006). Because a person may have a goal to accomplish a personal purpose it was important to find people who interpreted success through the completion of the task or goal as well as people who were motivated to complete the task only if it led to a much greater purpose. In order to have two different groups for comparison purposes, I selected a sample within each organization that included people who were considered by their supervisor as primarily goal/task driven as well as people who were considered purpose driven.

Very early on it became clear that when the only choice a supervisor was given to describe the primary driver was purpose or goal, the perception of purpose was more favorable. Only 30% of the people interviewed were identified as goal driven by their supervisor. One statement made during an interview reflected this negative connotation of being goal driven:

“I mean, not goal driven the way that some people are, and as an example meaning, I want to be worth this by a certain date or I want to have this kind of house by, or I have a Lamborghini by the time I am this age. I don't have goals that overt that I need to be—I need to have this particular title by a certain age. I don't have goals like that” 4-6.

Through the axial coding process, distinct themes emerged but the categorization of the responses did not always match up with the original classification of goal or purpose driven as provided by their supervisor. Saldana discusses allowing conceptual frameworks to emerge from within the data (Saldaña, 2013). It became apparent early on in the process of coding interviews that the way people responded to the first question was creating such a conceptual framework. The first question I asked the participants was, “What is important to you, motivates you to hop out of bed in the morning, and/or provides purpose or meaning to your life?” Two very distinct categories emerged. The phenomenon of the role personal purpose and personal goals had on symbiotic visions was clearly differentiated within two distinct areas of context, with the context being an individual's primary driver. This is supported by the statement that thematic sampling depends heavily on the quality of the data which is influenced by the setting or context (Boyatzis, 1998).

To eliminate the possibility of misclassifications by the supervisor due to possible negative connotations with being goal driven, and to avoid projecting, I identified a related and unbiased classification that could be applied to the subject's responses. Although I am really studying goals vs. purpose, the task vs. socio-emotional drivers construct (Boyatzis et al., 2014) is closely related and created an unbiased classification. This categorization was selected because it represents the contrast between being task or goal driven vs. future or purpose directed.

The way participants responded to the first question indicated their inclination for having task or socio-emotional/future oriented tendencies. Task-positive preferences are connected to being goal oriented and include a predilection for goal achievement, problem solving, decision making and the ability to control actions. Respondents were classified as task positive if their answers included being motivated by being acknowledged or appreciated for the work completed, maintaining balance, seeking opportunities, the work they do or solving problems. Socio-emotional preferences are much more future or purpose directed and are linked to social cognition, creativity and an openness to new ideas (Boyatzis et al., 2014). Respondents were classified as socio-emotional if their answers included being motivated by one's passion, making the world better, the desire to make a difference and helping others in need. Table 1 illustrates sample responses for each category.

**Table 1:** **Sample responses by group**.

Group 1—Task positive—11 respondents	Group 2—Socio-emotional—13 respondents
Responses indicating a Task Positive Nature	Responses indicating a Socio-emotional Nature
I love solving problems, I love taking things that are ambiguous and putting together a plan and attacking.	I get inspired by just making a difference. I love to engage.
Ultimately (I am) trying to find the right balance between work and life.	I am most excited and want to do more of hands-on connections with those who are in need.
My primary driver is—it's sort of self-absorbed and altruistic at the same time because I like being recognized and I like being appreciated.	I would say what gets me out of bed in the morning as far as employment goes is really understanding the long-term vision and believing in it and having a passion for it.
I think what drives me is a problem that doesn't have a solution. Ultimately, pulling resources and digging deep.	For me it was about the overall concepts, working for the greater good of something.

Eleven participants gave task positive responses and thirteen participants gave socio-emotional responses. One hundred percent of the participants who gave responses indicating socio-emotional preferences were perceived by their supervisor as being purpose driven. Yet only 55% of the participants who gave responses that would indicate a task positive preference were perceived as goal driven. When a participant gave more than one response, in all cases both responses fell into the same category. To assure I did not use the responses to this question in the coding process, the responses to this first question were removed from the coding process. The remainder of the questions were used for the qualitative analysis.

### Sample

Twenty-four people were interviewed; seven interviews from one company, six interviews from two companies and five interviews at a fourth company. The interviews averaged an hour in length. Because of the narrow focus of my study, after twenty two interviews no new themes were emerging and it was not necessary to expand the interview pool beyond 24.

Fourteen interviews were conducted face-to-face in a quiet location selected by the participant. Eleven interviews were conducted over the phone with the participant finding a quiet location that would allow them to reflect without interruption. IRB protocol was followed to assure consent, accuracy and confidentiality. Seventeen participants were based out of their corporate office and seven were based in other cities, outside their corporate headquarters location, around the United States. Participants were located in the states of Ohio, Illinois, Michigan, Massachusetts, New York and Wisconsin. After the interview, participants were categorized by industry, tenure at their current company, level within the company and gender, as illustrated in Table 2, to see if any of these descriptors impacted or contributed to the themes that emerged.

**Table 2:** **Descriptors applied to sample**.

**Industry sector**	**# of Participants**	**% of Total**
Food service	5	20.8
Oil and gas	6	25
Consumer goods	6	25
Distribution	7	29.2
**YEARS WITH COMPANY**	
1–5	5	20.8
6–10	10	41.7
11–15	4	16.7
16+	5	20.8
**LEVEL WITHIN THE COMPANY**	
Manager	7	29.2
Director	9	37.5
Sr. Leader	2	8.3
C-Suite	6	25
**GENDER**	
Female	12	50
Male	12	50

The interviews were coded using an open coding, exploratory approach as recommended by Saldaña (2013). I coded and sorted the interviews manually as well as electronically utilizing the web application Dedoose. After completing an initial coding on all interviews, interviews were reviewed to verify that consistent coding was applied. Focused coding was used to synthesize large sections of data (Glaser, 1978), such as stories, and axial coding was used to identify the frequency of common themes and the existence of dominant themes (Strauss, 1987). All coding was done blind to the initial criterion to see if themes emerged. The interviews were tracked with a two-digit identifier.

## Results

Four distinct themes emerged with a noticeable difference in how people responded based on their tendency to be task driven or socio-emotional: reference, motivational driver, temporal perspective and life/work integration vs. separation.

### Reference context—task driven people appear to be more self-referent whereas people that gravitate toward being socio-emotional appear to be more other-referent

One hundred percent of the respondents in the task positive group made one or more references to why something was important to, or impacted, them personally and/or how they put things through a self-referent lens before making decisions. Following are examples of responses that included a reference to themselves in how they choose to make decisions or validate a decision they had made.

“Sadly I didn't want the argument of having to deal with her being mad at me so I didn't do that” 3-6.

“It is something I like to do for myself” 3-5.

“I enjoy the leadership role not so much for the authority or for being in charge or whatever, but knowing that people are looking to me for direction” 1-7.

“How I validate myself, is my ability to help an organization get the information it needs and solve challenges” 4-5.

The socio-emotional group was more other-referent. Ninety-two percent of the respondents' comments were about how they are impacted by others and how their decisions are based on the needs or wants of other people.

“I always hope that I never put myself first, that is like the biggest thing for me is to always put other people first always, always, always” 4-1.

“And so when I see someone buzz through that and they think, wow, wait a minute, I just did this. I didn't think I could do it. What else can I do? And that gets me excited” 4-4.

“Another thing I guess I'd say I'm passionate about is getting people to be inspired to find whatever niche it is that they have the skill set and have the passion for” 2-5.

“I just made up my mind going home that day that I was not going to live a life where I couldn't really make an impact… the other thing that I really like about my job over the first 26 years is really helping people to get on a career path and make a difference in their life that way” 2-2.

“So I'm hoping that I'm able to inspire people to find what they can do within their own capabilities” 1-3.

One person in the socio-emotional group commented on how their personal needs were important but in the same sentence referenced the needs of others:

“I like to work so I can live. I think a balance is very important. I do enjoy working for a company that values people, I compare (my last company) and (my current company). At (my last company) when I first started, people were indeed resources, and resources are precious and you—you're trying to protect them. And over time, we had become assets and assets can be faded out and sold. And here, I think people are still the most important resource. And that's a really key part of the culture” 3-4.

A few of the statements from the self-referent group included others but in the same thought the person also referenced themselves. For example:

“I hate to ask for things. Like, as a single mom, I hate to ask for someone to watch my kids. I hate to have to say, can you please—would you help me. I know how that feels. And I'm sure that other people that are in need sometimes may not feel good about asking for help, but if you are showing them how to take care of themselves, everyone wants to be able to take care of themselves and have some dignity” 1-6.

Self-referent and other-referent comments were fairly equally distributed across all other descriptors.

### Primary motivational driver—individuals that are inclined to be more task oriented appear to be more motivated by the actual goal or milestone whereas socio-emotional individuals appear to focus on the purpose of the activity and the tasks or goals are only a means to a bigger end

Although both task positive and socio-emotional responses included the word “goal” in their vernacular, it was used differently. One hundred percent of the respondents that were categorized as task positive made references to being goal oriented or motivated by the accomplishment of the goal whereas 62% of the socio-emotional group referenced goals.

Goals create targets or align thought processes and vary in terms of the amount of specificity and time frame (Snyder, 2000). The task positive group appears to use goals more as the target or specific and measurable objective, whereas the socio-emotional group appears to use goals to align their activities toward a more holistic, far reaching purpose. Eighty-four percent of the socio-emotional group referenced or communicated a passion toward something that was very meaningful to them and 62% referenced a purpose that was driving their decisions. This compared to 18% that referenced a passion and 27% that made references to purpose in the task positive group. There was no significant variance across the descriptors.

The following statements are from individuals who tended to have a task orientation. They seem inclined to use goals to identify something that they felt they had the self-efficacy to accomplish, or that would give them a sense of completion and/or obligation.

“This is what I'm going to try to get done today” 3-5.

“There are times that I just, I can't wait to get to work. I can't wait to become engaged in a new problem. Where is the new problem?” 4-2.

“Well, I could – probably the most relevant one is the – is my goal to be home for dinner every night with my kids” 3-6.

“And so I think my goal – my job as his father is to help him – I don't want to feel guilty for the things he has, but I do want him to be grateful and by doing things for others it will actually demonstrate that higher purpose of like you know what, we do have – we have a responsibility as a matter of faith to do things for others” 3-6.

In contrast to this, the socio-emotional group appeared to use goals as steps in a process that were relevant to the extent that they provided guidance toward a greater purpose. This group was motivated by the bigger, overarching objective. The goals did not seem to be what drove their day-to-day activities or their decision making process. Responses that illustrate this are:

“I think you should have goals and think about the future. But I am fine doing a 180 any point in time you know, I am very comfortable doing that” 4-4.

“But I think my broader purpose is really helping find solutions to problems that actually work” 2-4.

“For me it was about the overall concepts, working for the greater good of something, and the lesson that I'm learning and the lessons that I can show my kid that, that was a greater benefit at the time, and I still very much would make the same decision. I think that that was more important than the financial aspect of the position” 2-5.

“Well, how can I then blend the personal goals that I have of making an impact on the world and like still have a job?” 2-4.

### Temporal perspective—there appears to be a noticeable difference in the temporal perspective of the decisions being made. decisions appeared to be made based on either the immediate task (transaction) or because the decision is foundational to transforming or achieving a greater, longer term objective

This is best illustrated by the proverb of giving someone a fish vs. teaching someone to fish—if you give someone a fish they'll eat for a day; if you teach someone to fish they'll eat for a lifetime. Tuckey, Bakker and Dollard credit Bass, Jung, Sosik, and Pearce for articulating the behavioral styles of transactional vs. transformational leadership. Transactional leadership is tied to motivating others through a direct relationship between the task and the reward, whereas transformational leadership is recognized as influencing, inspiring, and stimulating others (Tuckey et al., 2012). It is possible to extend this framework to decision making. Individuals in group 1 that are more prone to task positive tendencies, also appear to exhibit a more immediate and transactional perspective. They appear to make decisions based on having an impact on a case by case basis such that they can connect the activity to a reward. This group appears to demonstrate a desire to accomplish a goal because of the reward associated with its completion and getting satisfaction out of the task at hand. They referenced setting goals that were in the near future, a one-off that could stand alone and once accomplished, allowed them to move on to another goal. Sixty-four percent of the task positive oriented group articulated a preference for accomplishing a task that had definite completion criteria. Examples of task positive statements are as follows:

“What is the big, nasty problem that we are going to tackle today? It takes a lot of hard work but the success is so rewarding” 4-2.

“Well I mean at a very superficial level, having some success in my career has allowed me to provide for people” 4-5.

“I'm working with individuals and I can again align what they love and their passion and their strengths to what we need in the business, it's like the perfect marriage and it's, you know, I just have this great sense of satisfaction when I can make—I can help facilitate that process and I can help make that happen. It's very rewarding to me. I found great satisfaction in that” 3-3.

In contrast, 15% of the socio-emotional group talked about tasks that once finished could be considered complete, whereas 92% of this group spoke of their accomplishments as laying a foundation for a job that was far reaching and may not be completed by them personally. Often, they saw their role as inspiring others to take the lead, making a decision to do something based on the long-term implications. Individuals that are more socio-emotional in nature seem to exhibit behaviors and attitudes that are more transformational. They appear to be motivated to teach others to fish, creating a larger group of people who are working toward the same purpose. They appear to be driven to create a foundation for future progress, aligning people and inspiring people to transform something that is much bigger than themselves. For example:

“And so I want to be able to give them guidance, help maybe focus them, give them my experience, but ultimately empower them to really stretch and push them to go to places where they didn't think they could go before” 2-1.

“I want to live for all of us to get better and have better lives” 1-4.

“I think what drives who I am is the ability to help others get to where they – where they want to be” 4-3.

Males indicated a preference for having the end goal clear twice as often as females, but there did not appear to be much variance across other descriptors.

### Life work integration vs. separation—people associated with socio-emotional tendencies, appear to see a correlation between what they do at work and what they do outside of work more than task oriented individuals who appear to prefer separating their work from their personal life

Fifty-five percent of the task positive group were very clear that life and work were separate and 77% of the socio-emotional group were equally clear that they sought work/life integration. Seventy-seven percent of the socio-emotional group also indicated that what they did was very personal and meaningful to them. This contrasted to only 36% of the task positive group that made reference to what they do at work as being personal.

Examples of statements from the task positive group are:

“Even if I'm here longer, I still try to just – whatever I need to do, I do it here and I try not to do it at home” 3-5.

“Work-life balance is about one of the most important things we can strive for. And you know you hear a lot about work-life integration. I don't really believe in that” 3-1.

“I am very focused, intently focused on trying to get home in time for dinner so we can have a family-style dinner” 3-6.

Socio-emotional driven individuals appear to be driven to achieve a purpose that transcends the activities throughout their day, at work and at home, as demonstrated by the following quotes:

“So I can take my knowledge of sustainability and have it be part of who I am, way beyond the walls of (my current company)” 4-3.

“This, to me, was like a self-fulfillment thing. I can go to work and do what I do every day and make my income this way” 2-5.

“I played soccer, and so it's an inner-city program where they combine soccer with poetry and creative writing, which I love. And so I think I was able to see the connection. It's not just at work, but how you combine your life with something that you're—that you're doing on a day-to-day basis” 4-3.

“I felt like I was able to connect and preserve the best of the world, and do it for a living. It just felt like such a natural connection” 4-3.

## Discussion

I studied high performing leaders who worked for corporations that espoused a higher purpose vision. I found that leaders were able to articulate their purpose or meaning and they were able to express alignment of their purpose or personal goals with that of the organization. One of the key discoveries was that personal purpose and goals do appear to play a role in a symbiotic relationship with a company vision and one's articulation of how they are motivated and engaged—but in different ways depending on whether a person is primarily socio-emotional driven or task driven. It was observed that some of these executives had a deep and far reaching sense of purpose which seemed tied to driving the intent of the organization's higher purpose vision. When alignment was felt through the sense of the greater purpose, there was a symbiotic nature to this alignment; a deep, almost spiritual, commitment to deploying all aspects of their life, e.g., work and home, to making the world a better place and helping the organization be a contributing part of their personal purpose. Others had a task oriented way of describing their purpose which appeared more instrumental in helping move the organization toward its overall purpose through the achievement of goals and objectives. When alignment was felt through the organization's support of one's personal goals, there was a great sense of commitment, but a clear delineation between work and life ambitions.

This difference was found to influence how high-performing leaders were motivated to act and engage. Task-driven individuals were found to be more self-referent; motivated by the actual accomplishment of goals/milestones; more likely to make decisions based on being able to see the completion of the task in the near future; and clear that life and work were separate. Individuals who were socio-emotional in nature were found to be more other-oriented; more likely to focus on the greater purpose of an activity; more likely to make decisions with a long term, big picture in mind; and more likely to see their work as an integral vs. separate part of their life.

It is important to note that both groups spoke of the importance of connecting with others and relationships with people. Half of the task positive group made references to team identity whereas no one in the socio-emotional group spoke about the personal connections to a team. There is an opportunity to explore this deeper in future research.

### Reference context

The senior leaders interviewed gravitated toward either a self-referent or other-referent perspective which aligned with having task positive tendencies or socio-emotional tendencies, respectively. This primary reference appeared to impact their decisions around whether to act on and stay engaged in an activity. The tendency to gravitate toward a self-referent or an other-referent position may indicate that there are two different categorical perspectives of, or possibly a continuum from, a task to socio-emotional orientation that correlates to an individual's reference. Individuals that are more self-referent appear to frame their decisions and determine their desire to act and engage based on how it impacts, affects or connects to them, personally. Other-referent individuals appear to be more motivated to act or commit if the decision or objective is framed in relation to its impact on others.

Self-determination theory distinguishes autonomous and controlled motivation, both of which include extrinsic motivation. Autonomous motivation combines extrinsic and intrinsic factors that impact the identification of the activities value whereas controlled motivation is driven by extrinsic rewards or punishment; in both cases, they are in relation to one's sense of self (Deci and Ryan, 2008). Deci and Ryan (2000a) propose that self-determination is a basic need. This need is interpreted as a universal motivator such that by encouraging rewards and independent action, leaders can transfer control to followers and increase their self-determination and feelings of efficacy (Tuckey et al., 2012). My research opens the possibility that while increasing one's self determination through value and rewards that are meaningful to the individual may be very motivating for some, not all high performing senior leaders are motivated by self-rewards and self-value. The same company vision may be internalized differently based on one's primary reference. Self-determination theory is supported by the task positive group who appear to connect with personal value and rewards. Yet some people, as indicated by the socio-emotional group, may actually be more motivated if the message is put into the perspective of the greater good, or how they could inspire others, taking it out of a reward and self-value perspective.

### Primary motivational driver

Individuals that are inclined to be more task oriented appear to be more motivated by the actual goal or milestone, whereas socio-emotional individuals appear to focus on the purpose of the activity and the tasks or goals are only a means to a bigger objective. The task positive leaders appeared to analyze the measurable result or impact of the accomplishment of the goal to make their decisions regarding whether or not to proceed. The socio-emotional group seemed to focus on the impact it had on the more holistic, far reaching purpose.

In the task-positive network vs. the default mode network theory, Boyatzis et al. (2014), discovered a pull different than the traditional intrinsic and extrinsic tension. Their research points to an empathetic vs. analytical tension, indicating people are driven by emotional or cognitive reasoning. My second finding of a noticeable segregation of motivation between being task driven and socio-emotionally driven may support this theory; possibly adding an additional dimension. Some leaders appear to analytically evaluate the objective to determine their ability to accomplish the task at hand, whereas others emotionally evaluate their ability to inspire others to achieve a greater good. Individuals who are driven by the task appear to review the goal analytically, to determine the degree to which tangible acknowledgement of their accomplishments will come through via a sense of productivity or validation. Socio-emotional individuals appear to be passionate about the overarching purpose and motivated by something bigger than themselves, often other focused, which may not provide tangible or immediate results. For them, it is personal and they are motivated and engaged when they are able to inspire others to help make a difference in the overarching objective. It may be that this desire to help or inspire others to join a cause is motivated by empathy, in which case this task driver vs. purpose driver tendency supports the framework of the empathetic and analytical tensions. If the motivation is determined to be because of an alignment to a greater purpose—a goal with a longer perspective with measurements for success, albeit less obvious—this group may be processing their decisions through both an analytical and empathetic lens, possibly extending the framework of the empathetic and analytical tension theory. This data supports the need to have additional dimensions beyond the intrinsic and extrinsic tension such as the tensions proposed by Boyatzis et al. (2014), but more research would be require to understand if it also extends the framework.

### Temporal perspective

There appears to be a noticeable difference in the temporal perspective of the decisions being made by people who are task positive vs. socio-emotional. Decisions appear to be made based on either the immediate task (transaction) or because the decision is foundational to transforming or achieving a greater, longer term objective. This theme seems to impact why one makes the decisions he or she makes based on the locus of time used in reference to the goal or objective, e.g., one-off, task specific or a greater, longer term purpose orientation. This speaks to the time element that impacts the thought process behind the creation of goals referenced previously (Snyder, 2000). From the work on self-efficacy done by Bandura, people are motivated by the level of personal satisfaction they have around their ability to perform. This intrinsic motivation is sustained through the achievement of sub goals that connect to larger future goals (Bandura, 1977). Bandura is also referencing the temporal element and how it impacts the desire to accomplish tasks or sub goals in order to achieve the bigger objective.

The transactional vs. transformational leadership model has been used by practitioners to understand how to move their employees beyond a state of self-interest to a shared vision by providing meaning and purpose to their work. The underpinning is that a transformational leadership style uses inspiration through the connection to a higher purpose to motivate and engage followers to achieve a desired performance (Bass, 1990). The interpretation of my research indicates that this transformational leadership style may not always be better. The style of leadership best deployed may depend on where the individual falls on the continuum between being transactional or transformational. This supports the assertions summarized by Kowal Smith (2010) of Bass (1990), Walden et al. (1990), and Lowe et al. (1996) that the best leaders use both styles. Our possible contribution to this body of work is that a person who appears to be more transactional would prefer the aspirational vision to be translated into milestones, tasks or goals that, upon completion, would move the individual toward the desired end state. A transactional perspective's preference would be to clearly see the end goal on the horizon. A more transformational person would flourish under an inspirational approach of aligning around a meaningful purpose, and being given flexibility to work around, through or even without specific goals.

### Life work integration vs. separation

Task-positive and socio-emotional people seem to look at the integration or separation of work and life differently which appears to be connected to how they process decisions to act or engage. This finding seems to speak to the role their personal purpose or personal goals plays in creating alignment with that of the organization and how integrated their work is with the other aspects of their life. Dik and Duffy (2009) established the construct calling as including an external summons to a higher purpose and an alignment to a personal purpose that is other focused, or an advancement of a greater good. “A calling is a transcendent summons, experienced as originating beyond the self, to approach a particular life role in a manner oriented toward demonstrating or deriving a sense of purpose or meaningfulness and that holds other-oriented values and goals as primary sources of motivation” (Dik and Duffy, 2009, p. 427). One implication of their view is that individuals connect their work activity to their overall sense of purpose and meaning, or pursue careers that allow their work to be their calling. In either case, this implies an integration of work and life as one's vocation becomes a tool to accomplish their aspirational purpose. This desire to achieve a personal purpose creates motivation that extends beyond the office.

My research indicates that alignment to visions may exist in two very different ways, either as an alignment to a vision, or purpose, or support of personal goals. It may not be necessary to inspire people to see the greater good, or to stretch beyond themselves to be motivated, engaged and aligned with a higher purpose vision. Some people can have a personal purpose that is best described in a goal oriented fashion that is very instrumental in helping a company achieve its higher purpose vision, with a comfortable separation between work and life. At the end of the day, they will help the organization achieve its goal because they feel aligned to the company's vision even though they have a deep desire to keep their personal ambitions and the goals of the company separate.

## Implications for practice

People often choose a lower income to work in the not-for-profit world for personal reasons. This desire to be a part of something bigger, to make a difference, is something organizations may be able to tap into if their company vision is aligned with an employee's personal ambitions. Once this phenomenon is understood, it can be translated into a language that would help organizations understand how to harness this personal drive and intrinsic motivation.

The engagement and motivation of individuals who have a personal purpose or personal goals that can be accomplished through the work they do for an organization appears to be powerful and, once understood, could lead to a more flexible and personalized style of leadership. If an organization has a higher purpose vision that attracts employees with a vision symbiotic with that of the business, many of the traditional forms of external motivation may not be appropriate. By understanding why and what motivates task oriented vs. socio-emotional people to make decisions, a leader can apply the levers that trigger self-motivation instead of relying on a one size fits all.

A leader could increase motivation and engagement by providing alignment, resources and support in the form and perspective best understood and desired by each employee, then stepping away and allowing the employee to access and pull from the personal drive that comes from achieving their own aspirational purpose or goals in life. Similar to the conductor of an orchestra, the leader's primary duties would be to unify the organization, set the pace and tempo, and then listen, observe and direct as necessary to assure the organization is moving in the right direction. Employees who work for an organization that has a higher purpose, and are led by leaders who understand their drivers, can feel fulfilled and motivated as they use many of their waking hours to work toward their personal calling or purpose. At the end of the day, they can feel that their efforts were toward creating and supporting their ideal self, not the “ought self”, desired by someone else.

## Future research

This study was done within for-profit organizations, but the indications that symbiotic visions impact people in different ways is strong enough that it would be illuminating to do similar research in not-for-profit organizations to understand the similarities and differences.

This study illuminates the possible benefits of connecting to an individual's aspirational purpose or goals in a meaningful way through the related task positive vs. socio-emotional construct. Future research should be done specifically on goal vs. purpose drivers. To do this, clarification of the terms would need to be done to create unbiased and other determined classifications. Even with the limitations of this study, two categories emerged. Future research should be done on how to connect one's personal purpose and goals to a company's higher purpose and how to fit it into current and developing leadership theory.

There is work being done to understand what leaders need to do to motivate and engage their employees. Further research could be done to understand the correlation between leadership theories and the follower's response. The discovery of an analytical vs. empathetic tension is very important to understanding how followers make decisions. This research supports this theory and may be able to extend it to understand if this is a two dimensional framework or a continuum impacted by one's motivational driver. More research should be done to understand this relationship and the implications to leadership and communication styles.

Although both groups referenced the importance of relationships, 50% of the task focused group spoke about their identification with teams compared with no one in the socio-emotional group referencing teams or the personal identity associated with a team. Relationships were explored in the context of this study only to understand if they existed or were absent. Additional research would have to be done to understand the role team identities play in task-positive vs. socio-emotional decision styles.

### Limitations

Because this was an empirical study, possible meanings of these discoveries must be inferred in light of former research. Additional studies will be needed to truly comprehend and test these interpretations.

The study was exploratory in nature and designed to discover the role of personal purpose and personal goals when a symbiotic relationship with a higher purpose company vision and positive relationships existed or was absent. Therefore, interviews were conducted with senior leaders that were considered high performers in organizations that had a recognized higher purpose vision. The study does not consider what happens when high-performing employees have a personal purpose but work within an organization that does not have a higher purpose vision, or what happens when employees are not senior leaders in the organization nor are considered high-performers. More research would need to be done before this could be applied to the greater employee population.

This study does not assume that personal purpose is stable. Personal purpose and goals can evolve and change over time. This study only captures a point in time in which the senior leaders interviewed identified with the higher purpose vision of their organization.

This study does not imply that goal-driven or purpose-driven tendencies are good or bad, simply different drivers. I purposefully did not provide clear definitions of both terms prior to the interviews in order to not bias the selection of the participants or the direction of the responses to the questions. The apparent bias against being goal based and various understandings of the meaning of being purpose driven created a limitation. I mediated this limitation by using an already existing construct to categorize the themes that emerged based on Saldana's approach to allowing conceptual frameworks to emerge in the coding process and therefore, identifying groups associated with these frameworks (Saldaña, 2013). I used the first question to place people into groups that were defined by prior research on the topic of motivational drivers. I recommend that it be verified through another study that provides more clarification on the difference between the two drivers.

A fourth limitation is that all of the interviews were done with people in leadership positions. I recommend that a similar study be done of individuals not in leadership positions to understand if that variable impacts the findings.

## Conclusion

Purpose and goals appear to play a role in alignment to visions, motivation and engagement. High performing leaders are able to articulate and understand the symbiotic nature of their purpose or personal goals with that of the organization, yet how they frame their motivation and engagement is different depending on their orientation.

By understanding the roles personal purpose and goals play in alignment to company vision, motivation and engagement, a leader can apply the levers that trigger self-motivation. Additional research in this area could break the code to helping leaders increase motivation and engagement through alignment, and by providing resources and support in the form and perspective best understood and desired by each employee, allowing the leader to then step away as employees access and pull from the personal drive that comes from achieving their own aspirational purpose or goals in life.

### Conflict of interest statement

The author declares that the research was conducted in the absence of any commercial or financial relationships that could be construed as a potential conflict of interest.
