# Supplementary material for: The role of personal purpose and personal goals in symbiotic visions
Source: Front Psychol. 2015 Apr 14;6:443. doi: 10.3389/fpsyg.2015.00443 (PMC4396129; doi:10.3389/fpsyg.2015.00443)
Supplement: Supplementary file 1 [file DataSheet1.DOCX]

**APPENDIX**

**Interview questions**

1. What is important to you, what motivates you to hop out of bed in the morning, and/or what provides purpose or meaning to your life?
2. When did you discover this was important to you?
3. What about this inspires you, motivates you?
4. What makes you unhappy or disappointed?
5. Tell me how you connect this personal driver or calling to your life at home, at work and in your community.
6. Tell me about a time when you felt passionate about your work.
   1. What was it about that event that made you feel passionate?
   2. Did you feel supported? What does being supported feel like? Look like?
   3. What does your day look like when you are passionate, motivated and engaged? What does it feel like?
   4. What does your day look like when you are disengaged or unmotivated?
7. Tell me about a time when you felt what you do at work contributed to your personal purpose/ overarching goal.
8. How would you articulate the purpose/vision of your organization?
   1. How do they communicate their vision?
   2. What makes this vision believable?
   3. Tell me about a time when your personal purpose was very aligned with the organizations.
   4. How do you know when your personal purpose is aligned to that of your organization?
   5. What are the moments like when your personal purpose is not aligned with the organizations?
9. We are talking about personal purpose, goal, driver or a calling. What words would you use to describe this?
